# Supplementary material for: Immune-Related Adverse Events, Biomarkers of Systemic Inflammation, and Survival Outcomes in Patients Receiving Pembrolizumab for Non-Small-Cell Lung Cancer
Source: Cancers (Basel). 2023 Nov 21;15(23):5502. doi: 10.3390/cancers15235502 (PMC10705211; doi:10.3390/cancers15235502)
Supplement: Supplementary file 1 [file cancers-15-05502-s001.zip › cancers-2686970-supplementary.pdf]

	Progression-Free Survival (months)	Overall Survival (months)
	**Median (IQR)**	**Median (IQR)**
**Patients**		
**Without irAE**	3.6 (1.3-13.1)	7.8 (2.4-20.5)
**With irAE**	16.8 (5.9-n/r)	27.4 (13.9-n/r)
**irAE Events**		
**All**	n/a	n/a
**Dermatological**	19.6 (7.0-n/r)	26.9 (12.6-n/r)
**Endocrine**	17.1 (10.4-n/r)	33.6 (19.7-n/r)
**Gastrointestinal**	45.4 (24.2-n/r)	n/r (22.3-n/r)
**Hepatic**	11.5 (2.2-n/r)	n/r (12.0-n/r)
**Musculoskeletal**	19.4 (6.4-26.5)	19.9 (10.8-35.3)
**Pulmonary**	3.9 (0.4-7.5)	7.3 (0.7-10.7)

Supplementary Table S1: Progression-free survival and overall survival for patients with or without irAE and irAE events by the organ affected. n/r = not reached. n/a = not applicable

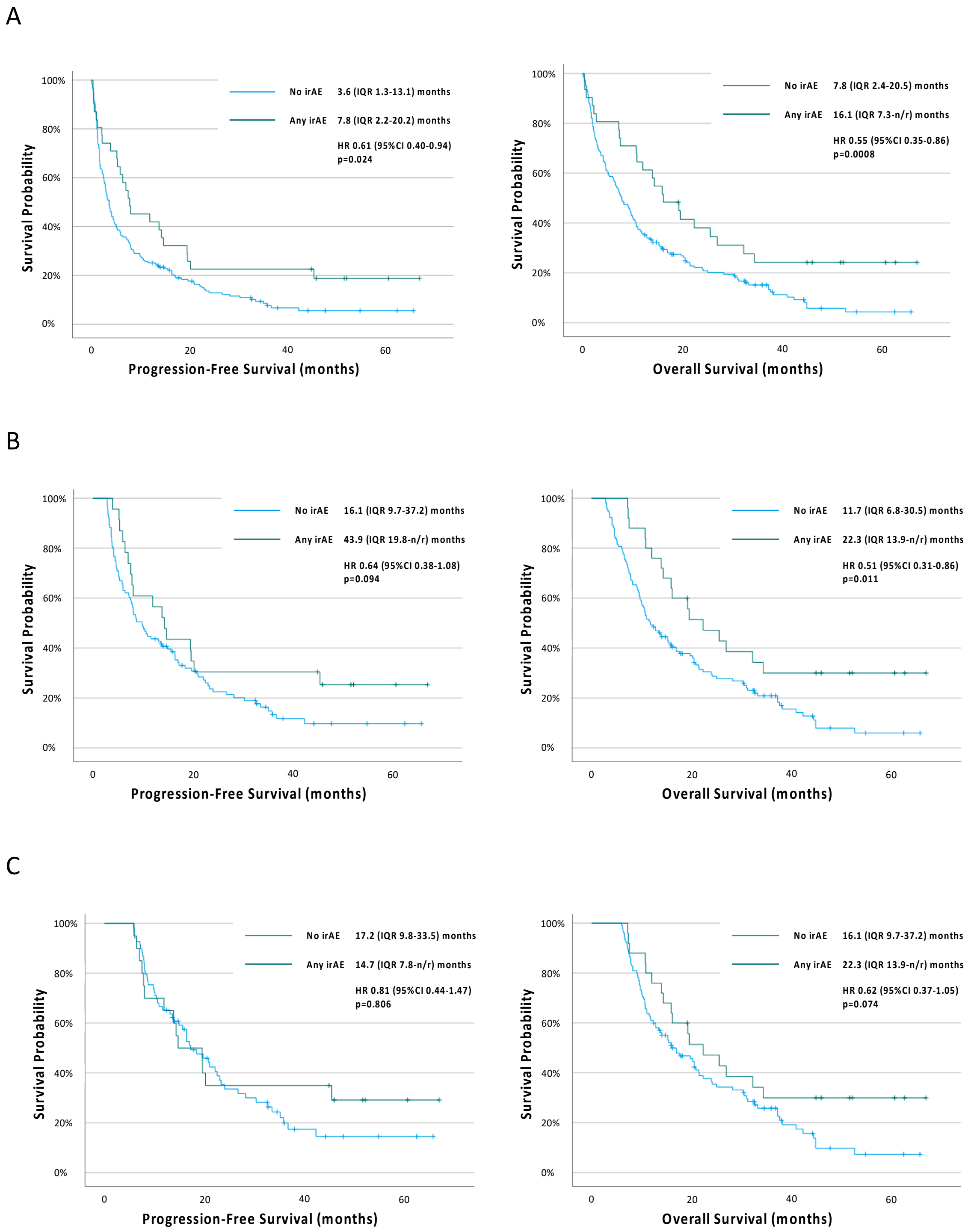

Supplementary Figure S1: Kaplan-Meier survival curves examining the relationship between the occurrence of severe (i.e. grade 3-5) immune-related adverse events and progression-free survival or overall survival in (A) all patients, (B) 12-week landmark and (C) 24-week landmark. n/r = not reached

A

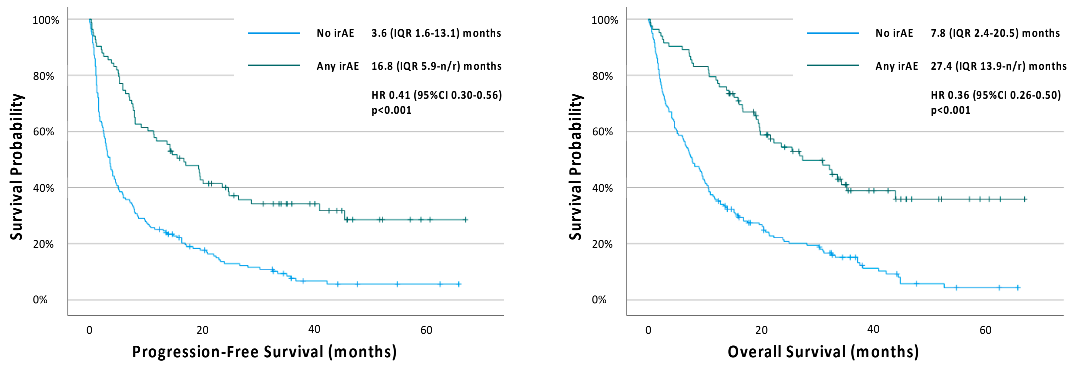

B

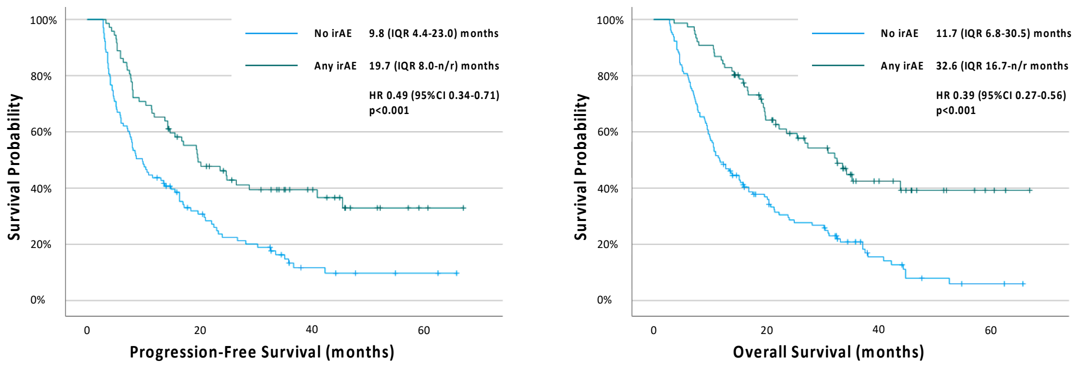

C

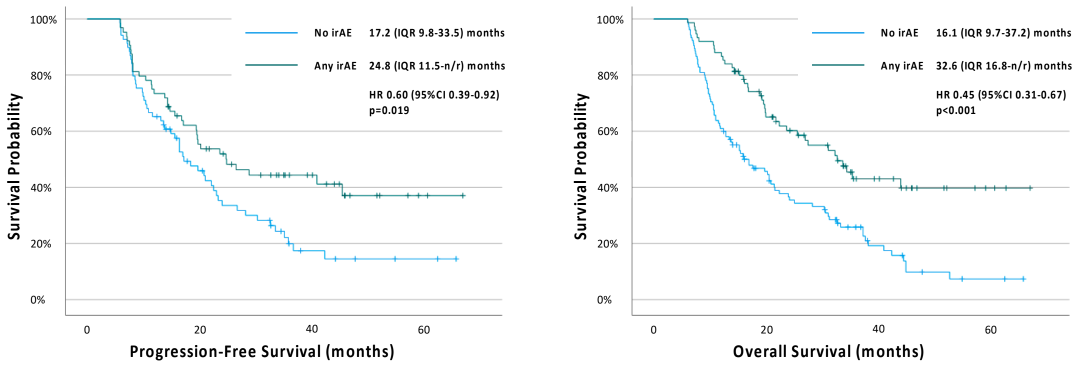

Supplementary Figure S2: Kaplan-Meier survival curves examining the relationship between the occurrence of mild (i.e. grade 1-2) immune-related adverse events and progression-free survival or overall survival in (A) all patients, (B) 12-week landmark and (C) 24-week landmark. n/r = not reached

A

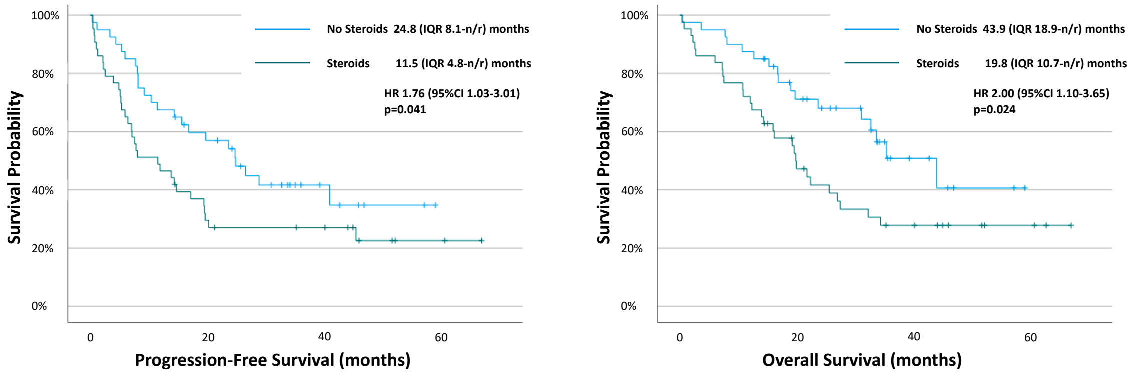

B

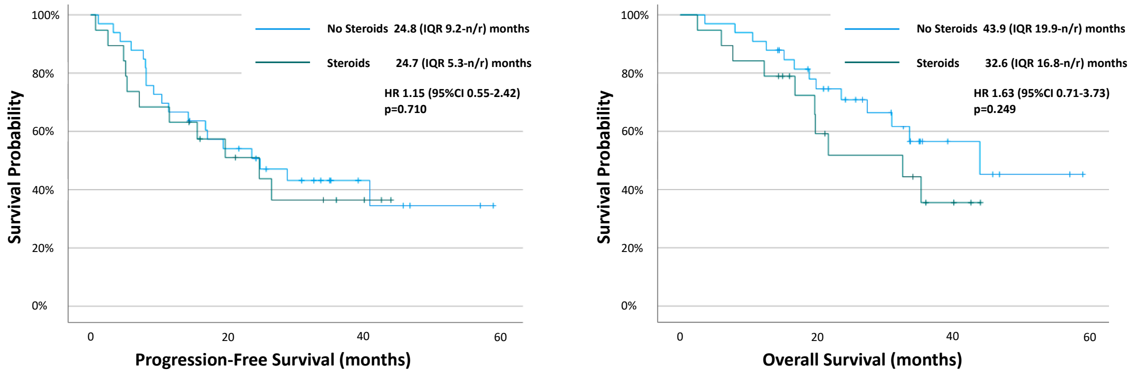

Supplementary Figure S3: Kaplan-Meier survival curves examining the relationship between steroid use and progression-free survival or overall survival in (A) all patients and (B) patients with mild (i.e. grade 1-2) immune-related adverse events. n/r = not reached

A

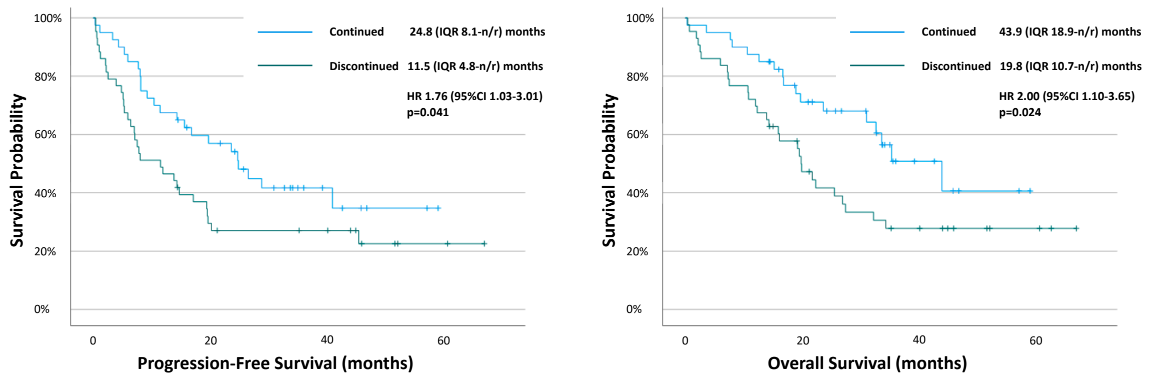

B

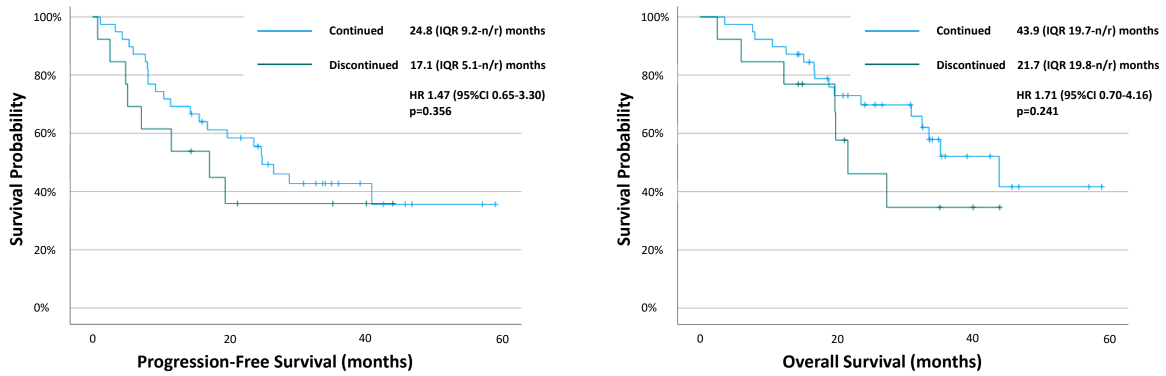

Supplementary Figure S4: Kaplan-Meier survival curves examining the relationship between treatment discontinuation and progression-free survival or overall survival in (A) all patients and (B) patients with mild (i.e. grade 1-2) immune-related adverse events. n/r = not reached

	Progression-Free Survival				Overall Survival			
	Univariate		Multivariate		Univariate		Multivariate	
	HR (95% CI)	*p*	HR (95% CI)	*P*	HR (95% CI)	*p*	HR (95% CI)	*p*
**Age (<75 years, ≥75 years)**	1.21 (0.85-1.73)	*0.288*			1.16 (0.79-1.70)	*0.447*		
**Sex (Male, Female)**	1.08 (0.83-1.41)	*0.574*			0.99 (0.75-1.30)	*0.924*		
**Performance Status (0, 1, 2)**	**1.55 (1.20-2.00)**	***0.01***	**1.40 (1.07-1.83)**	***0.012***	**1.64 (1.25-2.26)**	***<0.001***	***1.49 (1.12-1.98)***	***0.006***
**Histological Subtype (Squamous, non-squamous)**	1.06 (0.76-1.47)	*0.751*			1.07 (0.76-1.49)	*0.712*		
**PD-L1 Status (<90%, ≥90%)**	**0.76 (0.58-1.00)**	***0.048***	**0.71 (0.54-0.93)**	***0.012***	0.76 (0.58-1.01)	*0.055*		
**WCC (≤11x10^9/L , >11x10^9/L)**	**1.54 (1.17-2.03)**	***0.002***			**1.67 (1.26-2.23)**	***<0.001***		
**Neutrophils (≤7.5x10^9/L, >7.5x10^9/L)**	**1.76 (1.34-2.32)**	***<0.001***			**1.88 (1.42-2.49)**	***<0.001***		
**Neutrophil/Lymphocyte Ratio (≤5, >5)**	**1.68 (1.29-2.20)**	***<0.001***			**1.85 (1.40-2.44)**	***<0.001***		
**Platelet/Neutrophil Ratio (≤180, >180)**	1.27 (0.95-1.70)	*0.108*			**1.39 (1.02-1.88)**	***0.036***		
**Albumin (<35g/L, ≥35g/L)**	**1.93 (1.48-2.53)**	***<0.001***			**2.12 (1.60-2.80)**	***<0.001***		
**Prognostic Nutritional Index (<45, ≥45)**	**0.63 (0.48-0.83)**	***0.001***			**0.57 (0.43-0.76)**	***<0.001***		
**Scottish Inflammatory Prognostic Score (0, 1, 2)**	**1.66 (1.39-1.97)**	***<0.001***	**1.64 (1.38-1.96)**	***<0.001***	**1.75 (1.47-2.09)**	***<0.001***	**1.69 (1.41-2.02)**	**<0.001**

Supplementary Table S2: The relationship between prognostic factors and progression-free or overall survival in all patients. Bold/italics represent statistically significant findings (p<0.05)

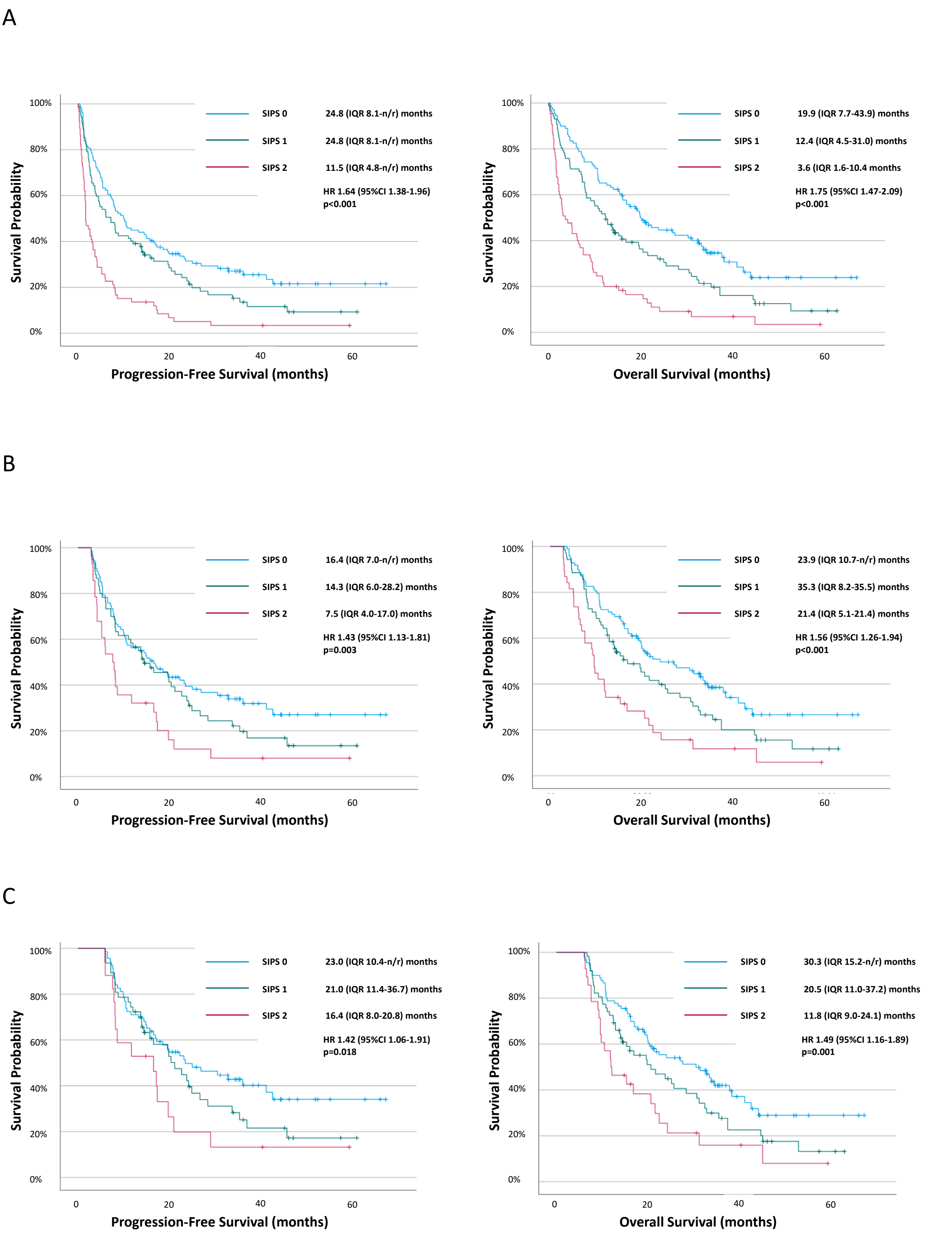

Supplementary Figure S5: Kaplan-Meier survival curves examining the relationship between the Scottish Inflammatory Prognostic Score (SIPS) and progression-free survival or overall survival in (A) all patients, (B) 12-week landmark and (C) 24-week landmark. n/r = not reached

A

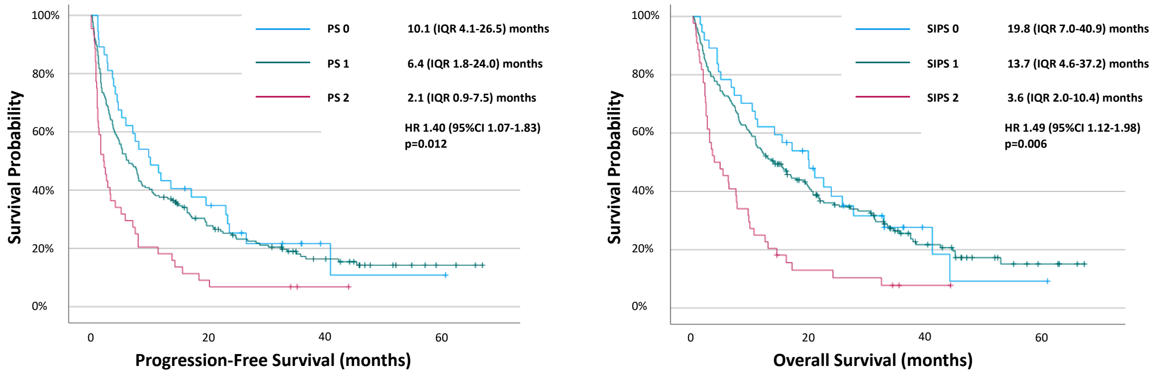

Supplementary Figure S6: Kaplan-Meier survival curves examining the relationship between performance status (PS) and progression-free survival or overall survival in all patients

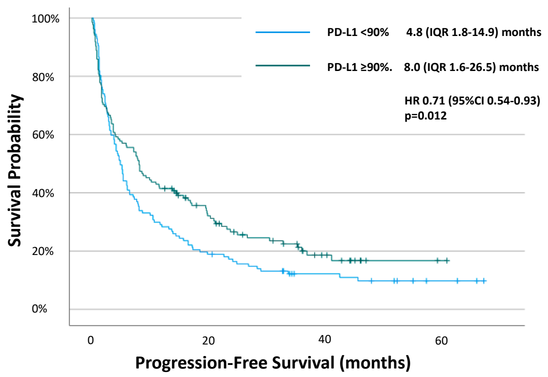

Supplementary Figure S7: Kaplan-Meier survival curves examining the relationship between PD-L1 status and progression-free survival
